# A Large-Scale Rheumatoid Arthritis Genetic Study Identifies Association at Chromosome 9q33.2

**DOI:** 10.1371/journal.pgen.1000107

**Published:** 2008-06-27

**Authors:** Monica Chang, Charles M. Rowland, Veronica E. Garcia, Steven J. Schrodi, Joseph J. Catanese, Annette H. M. van der Helm-van Mil, Kristin G. Ardlie, Christopher I. Amos, Lindsey A. Criswell, Daniel L. Kastner, Peter K. Gregersen, Fina A. S. Kurreeman, Rene E. M. Toes, Tom W. J. Huizinga, Michael F. Seldin, Ann B. Begovich

**Affiliations:** 1 Celera, Alameda, California, United States of America; 2 Leiden University Medical Centre, Leiden, The Netherlands; 3 SeraCare Life Sciences, Cambridge, Massachusetts, United States of America; 4 University of Texas, Houston, Texas, United States of America; 5 Rosalind Russell Medical Research Center for Arthritis, Department of Medicine, University of California San Francisco, San Francisco, California, United States of America; 6 National Institute of Health, Bethesda, Maryland, United States of America; 7 Feinstein Institute for Medical Research, North Shore L.I.J. Health System, Manhasset, New York, United States of America; 8 University of California Davis, Davis, California, United States of America; University of Oxford, United Kingdom

## Abstract

Rheumatoid arthritis (RA) is a chronic, systemic autoimmune disease affecting both joints and extra-articular tissues. Although some genetic risk factors for RA are well-established, most notably *HLA-DRB1* and *PTPN22*, these markers do not fully account for the observed heritability. To identify additional susceptibility loci, we carried out a multi-tiered, case-control association study, genotyping 25,966 putative functional SNPs in 475 white North American RA patients and 475 matched controls. Significant markers were genotyped in two additional, independent, white case-control sample sets (661 cases/1322 controls from North America and 596 cases/705 controls from The Netherlands) identifying a SNP, rs1953126, on chromosome 9q33.2 that was significantly associated with RA (OR_common_ = 1.28, trend P_comb_ = 1.45E-06). Through a comprehensive fine-scale-mapping SNP-selection procedure, 137 additional SNPs in a 668 kb region from *MEGF9* to *STOM* on 9q33.2 were chosen for follow-up genotyping in a staged-approach. Significant single marker results (P_comb_<0.01) spanned a large 525 kb region from *FBXW2* to *GSN*. However, a variety of analyses identified SNPs in a 70 kb region extending from the third intron of *PHF19* across *TRAF1* into the *TRAF1-C5* intergenic region, but excluding the *C5* coding region, as the most interesting (trend P_comb_: 1.45E-06 → 5.41E-09). The observed association patterns for these SNPs had heightened statistical significance and a higher degree of consistency across sample sets. In addition, the allele frequencies for these SNPs displayed reduced variability between control groups when compared to other SNPs. Lastly, in combination with the other two known genetic risk factors, *HLA-DRB1* and *PTPN22*, the variants reported here generate more than a 45-fold RA-risk differential.

## Author Summary

Rheumatoid arthritis (RA), a chronic autoimmune disorder affecting ∼1% of the population, is characterized by immune-cell–mediated destruction of the joint architecture. Gene–environment interactions are thought to underlie RA etiology. Variants within *HLA-DRB1* and the hematopoietic-specific phosphatase, *PTPN22*, are well established RA-susceptibility loci, and although other markers have been identified, they do not fully account for the disease heritability. To identify additional susceptibility alleles, we carried out a multi-tiered, case-control association study genotyping >25,000 putative functional SNPs; here we report our finding of RA-associated variants in chromosome 9q33.2. A detailed genetic analysis of this region, incorporating HapMap information, localizes the RA-susceptibility effects to a 70 kb region that includes a portion of *PHF19*, all of *TRAF1*, and the majority of the *TRAF1-C5* intergenic region, but excludes the *C5* coding region. In addition to providing new insights into underlying mechanism(s) of disease and suggesting novel therapeutic targets, these data provide the underpinnings of a genetic signature that may predict individuals at increased risk for developing RA. Indeed, initial analyses of three known genetic risk factors, *HLA*, *PTPN22*, and the chromosome 9q33.2 variants described here, suggest a >45-fold difference in RA risk depending on an individual's three-locus genotype.

## Introduction

Rheumatoid arthritis is the most common systemic autoimmune disease affecting approximately 1% of the adult population worldwide, with prevalence varying from 0.2–0.3% in East Asians to 6% in Pima and Chippewa Indians [1]. The disease is characterized by inflammation of the synovial tissue and local articular damage [2]. Disability in this inflammatory polyarthritis primarily stems from progressive bone erosion and comorbidity with coronary artery disease, infection and lymphoma [3],[4]. As with many other autoimmune conditions, RA affects women more commonly than men.

Although the etiology of RA is presently unknown, studies of RA heritability in two Northern European regions have demonstrated that an average of 60% of the disease variance can be attributed to genetic factors [5]. Through a combination of linkage and association studies, alleles segregating at the human leukocyte antigen (*HLA*) class II *DRB1* gene on chr 6p have consistently been shown to have strong RA-predisposing effects [6],[7]. That said, studies suggest that *HLA-DRB1* accounts for at most 50% of the phenotypic variance due to genetic effects [8]; therefore, loci not linked to the HLA region may play a crucial role in RA susceptibility.

Utilizing a variety of approaches such as positional mapping, candidate gene experiments and large-scale functional genetic association studies, several recent reports have yielded evidence for additional RA genes. The most robust, non-MHC, RA-associated marker is the R620W missense polymorphism in the *PTPN22* gene on chromosome 1p13, which has been repeatedly associated with RA in individuals of European ancestry [9]–[11]. In addition, positional cloning work has suggested the peptidyl arginine deiminase gene cluster (including *PADI4*) underneath a linkage peak on chr 1p36 may harbor susceptibility variants [12],[13] while well-powered association studies identified RA-associated SNPs in *STAT4* [14] and the *TNFAIP3* region [15],[16]. A promoter polymorphism of the Fc receptor-like 3 gene, *FCRL3*, and a SNP within the RUNX1 binding site of *SLC22A4* have also been implicated in RA susceptibility [17]–[19], both with conflicting reports [20],[21].

Interestingly, some of these disease-associated polymorphisms appear to have heterogeneity in effect sizes across ethnic groups; for example, the disease-associated variants in *PADI4* and *FCRL3* have a strong effect in East Asians but little effect in whites of European descent [10],[22]. Similarly, the *PTPN22* W620 risk allele is virtually absent in East Asians and therefore plays no role in RA risk in these populations [11]. As RA is a major cause of disability and is correlated with increased mortality in severe cases, genetic studies promise to improve public health. Importantly, as predicted by careful meta-analyses of linkage studies [23], some RA-susceptibility variants show pleiotropic effects across many autoimmune diseases [e.g. 11,14,24,25]. Consequently, further identification of RA genetic risk factors should aid in elucidating the underlying mechanisms of autoimmunity, in general, and may substantially impact drug discovery through the development of targeted diagnostics and therapeutics.

Arguing that the power of linkage disequilibrium-based designs to map disease alleles is high compared to other approaches, Jorde [26], Risch and Merikangas [27] and Long and colleagues [28] helped motivate the recent wave of successful genome-wide disease association studies. Propelled by technological developments, this shift has recently transformed common, complex disease gene mapping resulting in a number of convincing susceptibility variants [e.g. 29–31]. We took a large-scale candidate SNP association approach, very similar to that used in our recent study of psoriasis [32], to interrogate the genome for genetic variants that predispose individuals to RA. This genome-wide SNP panel (25,966 SNPs), which is primarily composed of missense (70%), acceptor/donor splice site and putative transcription-factor binding site SNPs, was applied to a multi-tiered, case-control association study of RA that incorporated replication of association effects as a key feature of the study design. By directly interrogating polymorphisms with higher likelihoods of producing biologically disruptive effects across multiple large sample sets, our aim was to maximize power to detect RA susceptibility genes.

We previously reported the identity of the RA-associated *PTPN22* R620W variant which was discovered in the first step (quality control of all DNA samples) of our RA scan [9],[33]. Here, we report our finding of variants in the *PHF19-TRAF1-C5* region on chromosome 9q33.2 that show strong and consistent association across three independent RA case-control studies (1732 cases/2502 controls), paralleling and extending the results of a whole-genome association study [34] and a candidate gene study [35]. Combining genetic information from *HLA*, *PTPN22* and *TRAF1* variants, we calculate the posterior probability of RA for every possible genotype combination. Results such as these may form the foundation for individualized prognosis and targeted medicine.

## Results

### Identification of the RA-Associated Chr 9q33.2 Region

We are conducting three sequential case-control studies to identify SNPs associated with RA. In the first study, DNA samples from white North Americans with (N = 475 cases) and without (N = 475 controls) RA (sample set 1, see Table 1 for a breakdown of the clinical characteristics of each sample set) were genotyped for a set of 25,966 gene-centric SNPs utilizing disease-phenotype-based pooled DNA samples (pooled DNA samples were used to economically increase genotyping throughput while minimizing DNA consumption). The allele frequency of each SNP was determined in cases and controls as described in the Methods and 1438 SNPs were significantly associated with RA using an allelic test (P<0.05); 88 of these SNPs mapped to chr 6p21 between *HLA-F* and *HLA-DPB1* within the major histocompatibility complex (MHC). Of the 1350 non-MHC SNPs, 1306 were evaluated in a second independent white North American sample set (661 cases and 1322 controls) by use of a similar pooling strategy (44 SNPs were not genotyped due to insufficient primer quantities). Eighty-nine statistically compelling SNPs (P_allelic_<0.05) with the same risk allele in these two sample sets were then individually genotyped in sample set 1 to verify the results from the pooled DNA phase of the experiment; 55 SNPs retained statistical significance (P_allelic_<0.05) and 44 have been individually genotyped in sample set 2. Twenty-eight of these were significant (P_allelic_<0.05) and are currently being evaluated in a third independent white Dutch sample set (596 cases and 705 controls).

**Table 1:** Demographic and clinical information.

	Sample Set		
Subphenotype	1^a^	2^b^	3^c^
Genetic background	White (North American)	White (North American)	White (Dutch)
No. of cases	475	661	596
No. of controls	475	1322	705
Female:male	314:161	536:125	362:196^d^
Average age of onset (years)	46.97±11.83	38.61±13.61	54.58±13.38^e^
% RF-positive	100%	82%	72%^f^

a 138 SNPs, including rs1953126, were genotyped in this sample set. Note, all 950 samples were genotyped for a single SNP, rs10818488, in the candidate gene study performed by Kurreeman et al [35].

b 73 SNPs, including rs1953126, were genotyped in this sample set. Note, 475 of these patient samples were included in the initial whole genome association study performed by Plenge et al [34].

c 43 SNPs, including rs1953126, were genotyped in this sample set. Note, 436 patients and 94 controls samples were included in the candidate gene study performed by Kurreeman et al [35].

d Information on gender was available for 558 patients.

e Information on age of onset was available for 306 patients.

f Information on RF status was available for 440 patients.

The most significant non-MHC SNP to emerge from a combined analysis of sample sets 1 and 2 after the *PTPN22* missense SNP, rs2476601 [9], was rs1953126 an intergenic SNP located 1 kb upstream of the human homologue to the Drosophila polycomblike protein-encoding gene, *PHF19*, on chr 9q33.2 near two excellent candidate genes, *TRAF1* and *C5* (individual genotyping: Sample Set 1: OR = 1.30, 95% CI 1.08–1.58, trend P = 0.007; Sample Set 2: OR = 1.35, 95% CI 1.18–1.56, trend P = 1.69E-05). This SNP was genotyped in sample set 3 showing a nonsignificant trend towards association: OR = 1.16, 95% CI 0.99–1.36, trend P = 0.066 (Table S1). No significant deviations from Hardy-Weinberg equilibrium were observed for the genotypes of this SNP in the cases or controls in the three sample sets. The frequency of the minor allele was approximately 30.8% in white North American controls increasing to 37.3% in white North American cases and 34.9% in Dutch controls increasing to 38.3% in Dutch cases. A combined analysis across all three sample sets was highly significant (OR = 1.28, 95% CI 1.16–1.40, trend P_comb_ = 1.45E-06).

### Chr 9q33.2 Fine-Mapping and LD Analyses

**Figure 1:**
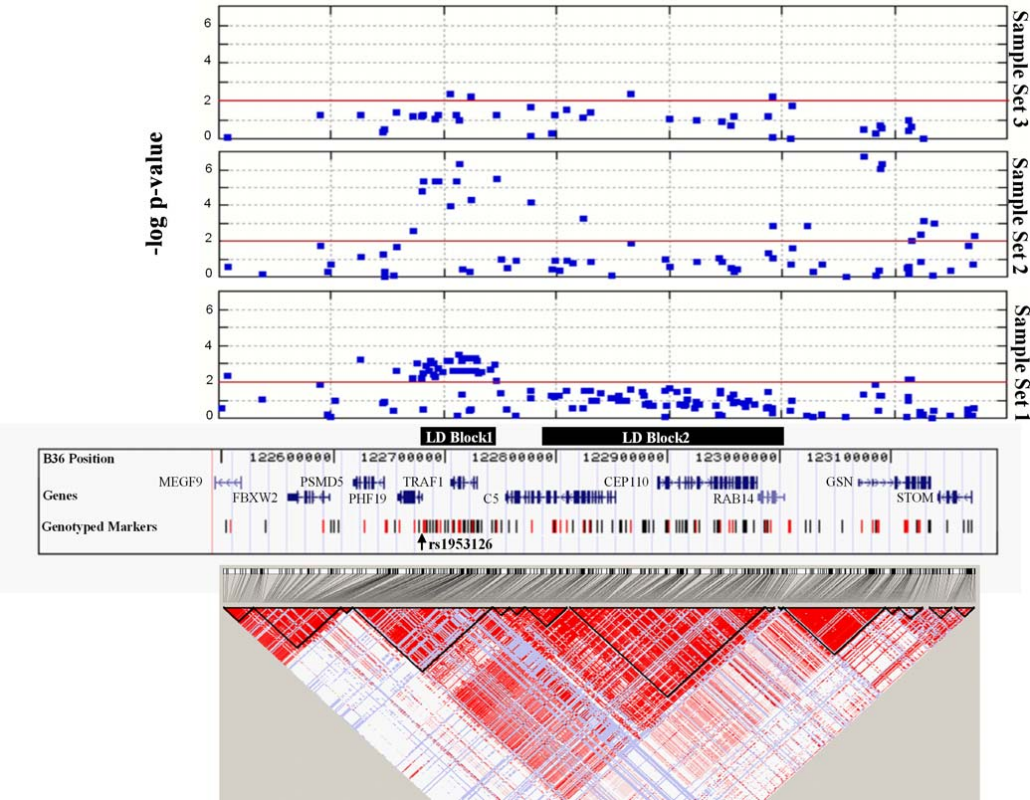
Case-control association results and linkage disequilibrium structure of the 9q33.2 region. A physical map of the 668 kb surrounding the original associated SNP, rs1953126, with the location of all 138 markers genotyped in sample set 1 noted. The markers in red indicate the 43 SNPs genotyped in all three sample sets. The locations of LD Block 1 and LD Block 2 are indicated. Above the physical map, the trend P-values are displayed for the SNPs genotyped in each of the three sample sets. The red line indicates trend P = 0.01. The LD structure across the 668 kb region from *MEGF9* to *STOM*, based on pairwise D' values from the CEU HapMap, is displayed below the physical map.

To further explore the association signal in this region, we used patterns of LD from the CEU HapMap data (www.hapmap.org) [36] to define a broad 668 kb region, extending from *MEGF9* to *STOM* on chr 9q33.2, for follow-up individual genotyping. Postulating two different disease models, one where the originally identified SNP, rs1953126, is in LD with one or more causative SNPs and a second model of allelic heterogeneity where several alleles at a locus independently predispose individuals to disease, we selected a combination of 137 LD and tagging SNPs from this region for follow-up genotyping in Sample Set 1 (Figure 1) (A detailed description of SNP selection is outlined in the Methods). Only four SNPs, all in the *RAB14-GSN-STOM* region, were mildly out of Hardy-Weinberg equilibrium (10^−4^<P<0.01) in the controls (Table S1). Including the original SNP, rs1953126, 38 of the 138 chr 9q33.2-region SNPs genotyped in Sample Set 1 were significant at the 0.01 level.

**Figure 2:**
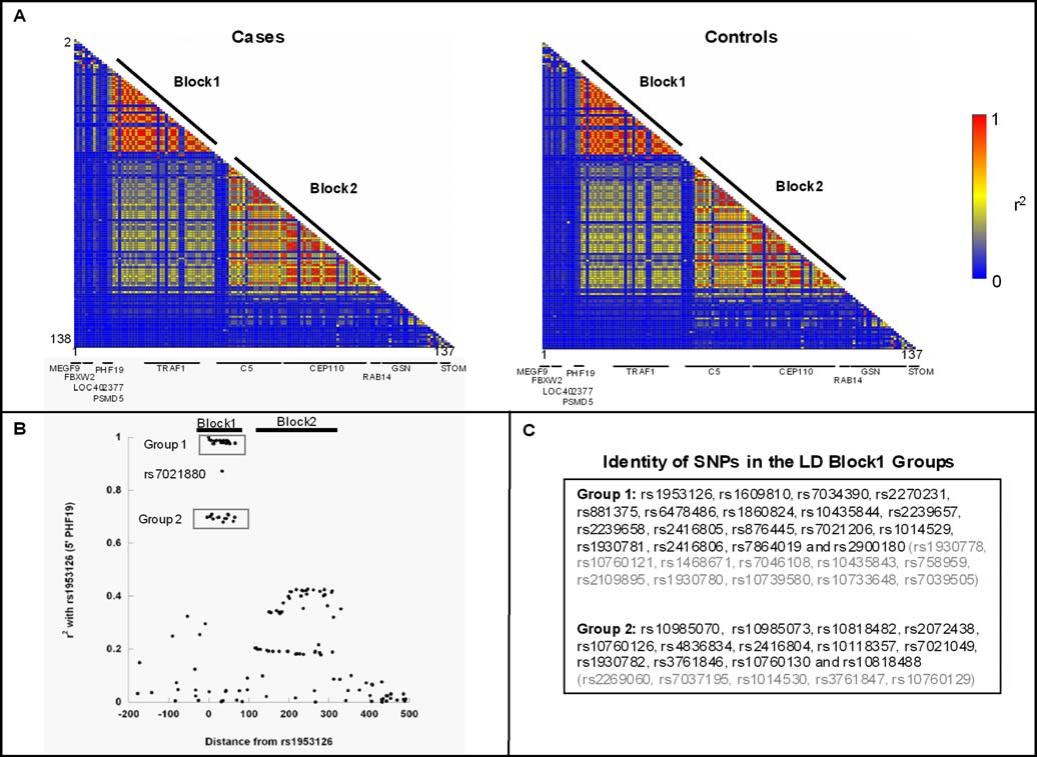
The LD architecture of the 9q33.2 region. (A) Pairwise linkage disequilibrium values (r^2^) for all 138 SNPs genotyped in Sample Set 1. Cases and controls are shown separately. (B) Pairwise LD values between rs1953126 and each of the 137 other SNPs genotyped in Sample Set 1. Locations of the two main LD blocks are shown in bold. (C) SNPs within each of the two LD groups in Block 1. SNPs in black were genotyped in this study and are listed according to their position. SNPs in grey were not genotyped but highly correlated (r^2^>0.93) with either Group 1 or Group 2 SNPs in the CEU HapMap data. Note that all of the SNPs in grey lie in LD Block 1 between rs10985070 and rs2900180.

To better understand these positive signals and select a subset of informative SNPs for genotyping in our other sample sets, we next investigated the LD architecture around rs1953126 by calculating pairwise r^2^ values for all 138 SNPs genotyped in Sample Set 1. Evaluating cases and controls separately revealed very similar LD patterns across this region (Figure 2A). There were two primary haplotype blocks (LD Block 1 and LD Block 2) (here an LD block is defined as a region in which over 75% of all pairwise r^2^ LD correlation values exceeded 0.3), with moderate LD between pairs of SNPs residing within each of the two blocks. LD Block 1, which contains the original SNP, rs1953126, and is approximately 70 kb, extends from rs10985070, an intronic SNP in the 5′ end of *PHF19*, across *TRAF1* into the *TRAF1-C5* intergenic region to rs2900180. Approximately 214 kb in length, LD Block 2 ranges from the middle of *C5* to the *RAB14-GSN* intergenic region. Given that haplotype block structures can have complex LD patterns within and between blocks and that we were focused on a single associated SNP in this region (rs1953126), we present a higher resolution plot shown in Figure 2B where pairwise r^2^ values were calculated for rs1953126 and each of the remaining 137 SNPs, revealing groups of highly correlated SNPs not readily visible in the LD heat-map.

Integrating the Sample Set 1 association results with the LD measures, we found that the original SNP, rs1953126, was highly correlated (r^2^>0.95) with 17 other SNPs (Group 1 in Figures 2Band 2C in LD Block 1. As predicted, these 18 SNPs have similar association results increasing in frequency from approximately 30–31% in controls to 36–37% in cases (OR = 1.29–1.35, trend P∼0.002–0.009) (Table S1). Of interest was the observation that 20 non-Group 1 SNPs were associated with disease at equal or greater significance including 14 other SNPs from LD Block 1. Thirteen of these other LD Block 1 SNPs, which were highly correlated with one another (r^2^>0.95) (Group 2 in Figures 2Band 2C and reasonably correlated with the Group 1 SNPs (r^2^ = 0.66–0.72), had minor allele frequencies of approximately 38% in controls increasing to 46% in cases (OR = 1.34–1.39, trend P≤0.002). The fourteenth significant SNP in LD Block 1, rs7021880, a *TRAF1* intronic SNP, was also highly significant (OR = 1.43, trend P = 3.12E-04) increasing in frequency from 27.1% in controls to 34.7% in cases. This SNP was in LD with both Group 1 (r^2^ = 0.82–0.90) and Group 2 (r^2^ = 0.59–0.64) SNPs (Figure 2B). The six other SNPs with P values <0.01 lie upstream of LD Block 1 (n = 4) or downstream of LD Block 2 in *GSN* (n = 2) (Figure 1, Table S1) and, with the exception of the *PSMD5* intronic SNP rs10760117, were not as significant as many of the LD Block 1 SNPs.

Given the association results and the LD structure, we selected 72 of the 137 fine-scale mapping SNPs to genotype in Sample Set 2 (661 white North American RA patients and 1322 matched white North American controls) (Table S1). This subset of fine-scale mapping SNPs was chosen to reduce the genotyping load, while capturing the association signals and retaining full coverage of the genetic variation in this region. Two of these 72 SNPs, rs12683062 (in *CEP110*) in the cases and rs9409230 (a *RAB14-GSN* intergenic SNP) in the controls, were moderately out of Hardy-Weinberg equilibrium (P = 2.56E-04 and P = 0.003, respectively; Table S1). Including the original SNP, rs1953126, 23 of these 72 SNPs were significant (trend P<0.01) in Sample Set 2; however, the nine significant LD Block 1 SNPs in Sample Set 1 were the most significant, replicated SNPs in Sample Set 2 (Figure 1). Interestingly there were three SNPs in *GSN* (rs10985196, rs7046030 and rs12683459), all highly correlated with pairwise r^2^ values >0.90, which were highly significant (trend P<10^−6^) in Sample Set 2 but only marginally significant in Sample Set 1 (trend P = 0.01–0.05). The difference between the two sample sets appears to be the result of disparate control allele frequencies – the case allele frequencies are nearly identical between the two sample sets (∼22%) but the control allele frequencies differ by 3% (18–19% in Sample Set 1 vs 15.5–16.5% in Sample Set 2) (Table S1).

Forty-two SNPs were genotyped in Sample Set 3 (596 white Dutch RA patients and 705 white Dutch controls); none of these SNPs rejected HWE at the P<0.01 significance level (Table S1). These 42 SNPs span over 600 kb and were selected to cover genetic variability, association patterns and gene boundaries. Four of the 42 SNPs, spanning 286 kb from *TRAF1* to *RAB14*, were significant at the 0.01 level (Figure 1). Of these four, two SNPs (rs4836834 and rs7021049) were members of Group 2 from LD Block 1, perfectly correlated (r^2^ = 1) and both SNPs were highly significant in all three sample sets. The other two significant SNPs, rs1323472 and rs942152, were only moderately if at all significant in Sample Sets 1 and 2. The six Group 1 SNPs genotyped in Sample Set 3 were close to the 0.05 significance level, with the most significant of these being the synonymous P340P *TRAF1* SNP, rs2239657 and the *TRAF1-C5* intergenic SNP, rs2900180 (trend P = 0.052) (Table S1). The *TRAF1* intronic SNP, rs7021880, was not significant in this sample set (trend P = 0.102).

In a combined analysis of the 43 SNPs genotyped in all three sample sets, including the original SNP, rs1953126, 25 SNPs, spanning a region of over 525 kb from rs7026635 within *FBXW2* to rs10818527 within *GSN*, were significantly associated with RA (trend P_comb_<0.01) (Table 2). Several of these SNPs exhibited consistent and strong association across all three sample sets (Table S1). Using either a combined trend or genotypic P-value, the top-ranked five SNPs were: rs6478486, rs4836834, rs2239657, rs7021880 and rs7021049 (listed in order of position). All reside within or near *TRAF1* in LD Block 1, had common odds ratios of approximately 1.3 and were highly significant (trend P_comb_<1.5E-07) (Table 2).

**Table 2:** Combined analysis of 43 chr 9q33.2 SNPs genotyped in all three RA sample sets.

				Combined Analysis		
Marker	Gene	Type	Position & Alleles^a^	OR_common_ (95% CI)^b^	Trend P_comb_ c	Genotypic P_comb_ c
rs10760112	*MEGF9*	intronic	C122507391T	1.17 (1.02–1.23)	0.035	0.136
rs7026635	*FBXW2*	intronic	G122589848A	1.24 (1.10–1.35)	0.001	0.012
rs10760117	*PSMD5*	intronic	T122626558G	1.26 (1.10–1.31)	2.79E-04	0.003
rs10739575			G122645922A	1.16 (1.03–1.30)	0.081	0.349
rs933003			A122647650G	1.12 (0.79–1.40)	0.255	0.243
rs1837	*PHF19*	3′UTR	T122658050C	1.28 (1.12–1.36)	2.17E-04	0.002
rs1056567	*PHF19*	S181S	T122671866C	1.25 (1.12–1.35)	1.11E-04	0.002
rs1953126			T122680321C	1.28 (1.16–1.40)	1.45E-06	4.24E-05
rs1609810			G122682172A	1.29 (1.19–1.42)	1.92E-07	5.24E-06
rs881375			T122692719C	1.27 (1.17–1.41)	4.69E-07	1.09E-05
rs6478486			T122695150C	1.29 (1.19–1.42)	1.35E-07	3.75E-06
rs4836834	*TRAF1*	3′UTR	T122705722A	1.32 (1.19–1.43)	8.13E-08	1.84E-06
rs2239657	*TRAF1*	P340P	G122711341A	1.29 (1.19–1.43)	1.49E-07	3.89E-06
rs7021880	*TRAF1*	intronic	C122713711G	1.33(1.21–1.46)	5.41E-09	2.27E-07
rs7021049	*TRAF1*	intronic	G122723803T	1.32 (1.20–1.43)	4.09E-08	1.22E-06
rs2900180			T122746203C	1.27 (1.18–1.41)	3.32E-07	7.62E-06
rs2269066	*C5*	intronic	T122776839C	1.29 (1.14–1.53)	1.68E-04	0.001
rs2269067	*C5*	intronic	C122776861G	1.27 (1.17–1.46)	1.71E-05	1.04E-04
rs2159776	*C5*	intronic	C122795981T	1.11 (0.99–1.19)	0.190	0.135
rs7040033	*C5*	intronic	A122798865G	0.86 (0.80–0.96)	0.018	0.060
rs17611	*C5*	I802V	A122809021G	0.84 (0.79–0.94)	0.006	0.040
rs10985126	*C5*	G385G	C122823755T	1.20 (1.11–1.39)	8.69E-04	0.001
rs2416811	*C5*	intronic	T122829455C	0.85 (0.79–0.95)	0.008	0.023
rs1323472			C122866156G	1.23 (1.12–1.34)	1.57E-04	7.06E-04
rs9657673	*CEP110*	intronic	T122900196C	0.86 (0.81–0.96)	0.019	0.052
rs10081760	*CEP110*	intronic	A122924127G	1.15 (1.03–1.25)	0.049	0.066
rs12683062	*CEP110*	intronic	T122946625G	1.12 (1.00–1.33)	0.209	0.029
rs3747843	*CEP110*	intronic	A122954127G	1.13 (1.01–1.21)	0.108	0.304
rs3736855	*CEP110*	V1398V	A122956841T	0.87 (0.82–0.98)	0.048	0.191
rs10760152	*RAB14*	intronic	A122987806C	1.15 (1.05–1.27)	0.028	0.024
rs942152	*RAB14*	intronic	C122991506T	1.18 (1.11–1.32)	2.53E-04	0.002
rs9408928	*RAB14*	intronic	C122991738T	1.11 (0.93–1.38)	0.364	0.378
rs9409230			T123007581A	1.14 (0.93–1.40)	0.499	0.217
rs7030849			C123009655T	1.18 (1.08–1.29)	0.003	0.014
rs10985196	*GSN*	intronic	A123072865C	1.25 (1.18–1.46)	6.33E-07	4.12E-06
rs306781	*GSN*	intronic	C123082765T	0.68 (0.59–1.16)	0.119	0.284
rs7046030	*GSN*	intronic	C123087058T	1.26 (1.18–1.47)	2.05E-06	1.99E-05
rs12683459	*GSN*	intronic	A123088119G	1.25 (1.18–1.47)	1.36E-06	9.79E-06
rs4837839	*GSN*	intronic	T123111948C	0.85 (0.82–0.97)	0.021	0.076
rs306783	*GSN*	intronic	T123112418C	1.11 (1.00–1.19)	0.198	0.405
rs306784	*GSN*	intronic	T123112473G	1.15 (1.03–1.24)	0.049	0.131
rs10818527	*GSN*	intronic	A123115075G	1.21 (1.08–1.31)	0.001	0.004
rs12683989	*GSN*	intronic	T123125867C	1.17 (1.05–1.50)	0.016	0.010

a Positions according to genomic contig NT_008470.18 (Entrez Nucleotide). The minor allele is listed first, followed by the position in National Center for Biotechnology Information Genome Build 36.2 and then the major allele.

b Calculated for the minor allele using a Mantel-Haenszel common OR.

c Calculated using Fisher's combined test.

### Multiple Testing

Since false-positive results can be problematic in any large-scale experiment in which modest nominal significance levels are used, we corrected the results from the combined analysis for multiple testing using the method of Dunn-Sidak [37]. Seven SNPS, all within LD Block 1, survived a Dunn-Sidak correction for 25,966 SNPs at P<0.01. The corrected trend P_comb_ values for the five most significant SNPs were: 0.004 for rs6478486 and rs223957 (Group 1), 0.002 for rs4836834 and 0.001 for rs7021049 (Group 2), and 1.3E-04 for rs7021880.

### Haplotype Sliding Window

**Figure 3:**
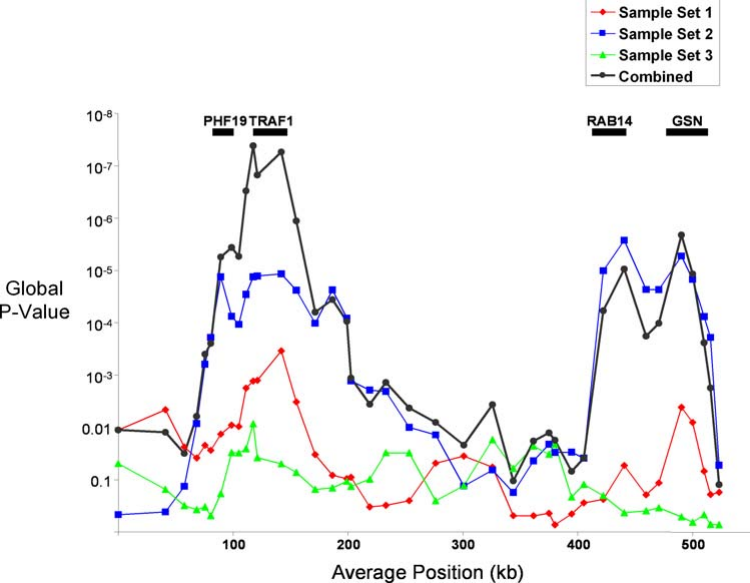
A five-SNP sliding window haplotype analysis of the 9q33.2 region. Each sample set is shown separately with the combined analysis in bolded black. The approximate location of the *PHF19*, *TRAF1*, *RAB14* and *GSN* genes are listed above.

Given that our fine-scale-mapping SNPs cluster into various groups based on their pairwise r^2^ values and that under many models haplotypes can be more informative than single-markers [38], we used the Haplo-Stats package [39] to run a 5-SNP sliding-window haplotype association analysis on the 43 SNPs genotyped in all three sample sets separately for each sample set and then combined the statistical evidence across all three sample sets. The combined analysis revealed a 29 kb-wide maximum peak of global association for haplotypes comprised of alleles segregating at rs6478486-rs4836834-rs2239657-rs7021880-rs7021049 in LD Block 1 (P_comb_ = 4.15E-08) (Figure 3). This region ranges from 9 kb downstream of *TRAF1* in the *PHF19-TRAF1* intergenic region to intron 3 within *TRAF1*. Aside from this peak and a second highly significant peak in the *TRAF1* region (P_comb_ = 5.45E-08; rs2239657-rs7021880-rs7021049-rs2900180-rs2269066), a second region of interest was centered over the *RAB14-GSN* region (P = 2.11E-06).

Of these two regions, we view the disease association evidence to be stronger for the *PHF19-TRAF1* region for several reasons: First, combined analyses across all studies yielded the most significant results for both single markers and haplotypes in this region. Second, the association signal at this region shows a higher degree of consistency across the three studies. Indeed, Sample Set 3 haplotypes in the *RAB14-GSN* region show very little deviation from the null hypothesis (Figure 3). Finally, as discussed above, a subset of SNPs in the *RAB14*-*GSN* region (e.g. rs10985196, rs7046030, rs12683459) displayed substantial differences in control allele frequencies between the two North American groups (Table S1) drawing into question the validity of the association results for these SNPs.

### Haplotype Analyses of LD Block 1 Variants

While both the single marker and sliding window haplotype analyses pointed to LD Block 1 as harboring RA-associated SNPs, these analyses did not identify a single SNP that was clearly the most significant across all three studies. The *TRAF1* intronic SNP, rs7021880, was the most significant SNP in Sample Sets 1 (trend P = 3.12E-04) and 2 (trend P = 5.09E-07) and in the combined analysis (trend P_comb_ = 5.41E-09); however, this SNP was not significant in the Dutch sample set (trend P = 0.102) where the Group 2 SNPs, rs4836834 and rs7021049, were the most significant (trend P = 0.004 and 0.006, respectively) (Tables S1 and S2). Interestingly, these Group 2 SNPs ranked second in significance in Sample Set 1 and in the combined analysis while in Sample Set 2 they ranked third behind rs7021880 and the Group 1 SNPs.

Given these results, we analyzed the haplotype structure of LD Block 1 using a subset of the nine SNPs from this region genotyped in all three studies. Taking into account the LD structure we picked rs2239657, the P340P *TRAF1* synonymous polymorphism to represent the six Group 1 SNPs; rs7021049, a *TRAF1* intronic SNP to represent the two Group 2 SNPs; and rs7021880 for these analyses. Haplotype frequencies for these three SNPs were estimated using the Haplo.Stats package [39] revealing the same four common haplotypes in each study (Table 3). Two of these haplotypes, AGT and GCG were strongly associated with disease (P_comb_ =  3.08E-08 and 8.00E-09, respectively), with the former being protective – decreasing in frequency from ∼60.9% in North American controls to 53.8% in North American cases and 56.7% in Dutch controls to 51.2% in Dutch cases (OR_common_ = 0.76, 95% CI 0.70–0.83); and the latter susceptible – 27.0% in North American controls increasing to 34.7% in North American cases and 33.2% in Dutch controls increasing to 36.0% in Dutch cases (OR_common_ = 1.32, 95% CI 1.21–1.45). These haplotype P_comb_-values were not significantly different from those calculated for the individual SNPs (Table 2) suggesting there is no strong evidence for synergistic cis-acting effects between these variants.

**Table 3:** Three SNP haplotypes for LD Block 1.

	Sample Set 1				Sample Set 2				Sample Set 3				Combined	
	Global P = 6.00E-04^a^				Global P = 3.77E-05^a^				Global P = 0.033^a^				Global P_comb_ = 1.81E-07^b^	
	No. (Frequency) in				No. (Frequency) in				No. (Frequency) in					
Haplotype^c^	Case	Control	P	OR	Case	Control	P	OR	Case	Control	P	OR	P_comb_ d	OR_common_ (95% CI)^e^
AGT	507(0.539)	582(0.619)	5.08E-04	0.72	708(0.537)	1595(0.605)	4.01E-05	0.76	604(0.512)	794(0.567)	0.005	0.80	3.08E-08	0.76 (0.70–0.83)
GCG	326(0.347)	253(0.269)	2.13E-04	1.44	457(0.347)	714(0.271)	8.71E-07	1.43	425(0.360)	465(0.332)	0.133	1.13	8.00E-09	1.32 (1.21–1.45)
AGG	85(0.090)	71(0.075)	0.250	1.22	108(0.082)	232(0.088)	0.540	0.93	122(0.103)	120(0.086)	0.127	1.22	NC	1.09 (0.93–1.27)
GGG	22(0.023)	32(0.034)	0.168	0.68	41(0.031)	92(0.035)	0.539	0.89	25(0.021)	20(0.014)	0.135	1.49	NC	0.93 (0.70–1.21)
Other	0	2(0.002)			4(0.003)	3(0.001)			5(0.004)	1(0.001)				

a The Haplo.Stats package was used to test for association between haplotypes and disease status.

b Calculated with use of Fisher's combined test.

c These haplotypes consist of SNPs: rs2239657, rs7021880 and rs7021049 respectively.

d Calculated for haplotypes with the same effect (risk or protection) in all three sample sets, with use of Fisher's combined test.

e Mantel-Haenszel common odds ratio with confidence intervals from Monte Carlo simulation.

### Dosage Effects

To explore the effect of the number of copies of each haplotype at these three sites (rs2239657, rs7021880 and rs7021049) along with any dominant/recessive effects between haplotypes, we estimated diplotypes using the pseudo-Gibbs sampling algorithm from the program SNPAnalyzer [40]. Analyzing the diplotypes individually, two diplotype combinations achieved statistical significance (P<0.01) when compared to all other diplotypes (Table 4). The AGT/AGT diplotype was strongly associated with protection against RA (OR_Common_ = 0.68, 95%CI 0.59–0.78; P_Comb_ = 5.35E-07), whereas the less frequent GCG/GCG diplotype was associated with predisposition (OR_Common_ = 1.42, 95%CI 1.16–1.75; P_Comb_ = 0.005).

**Table 4:** Diplotype Analysis for the TRAF1-region SNPs rs2239657, rs7021880 and rs7021049.

	Sample Set 1 Global P = 0.0069^a^				Sample Set 2 Global P = 1.3E-04^a^				Sample Set 3 Global P = 0.058^a^				Combined Analysis Global P_comb_ = 8.22E-06^b^	
	No. (Frequency) in				No. (Frequency) in				No. (Frequency) in					
Diplotype^c^	Case	Control	P^d^	OR	Case	Control	P^d^	OR	Case	Control	P^d^	OR	P_comb_ e	OR_common_ f (95%CI)
AGT/AGT	140(0.297)	180(0.383)	0.006	0.68	183(0.278)	482(0.366)	8.21E-05	0.67	153(0.259)	232(0.331)	0.005	0.70	5.35E-07	0.68(0.59–0.78)
AGT/Other	51(0.108)	64(0.136)	0.197	0.77	86(0.131)	204(0.155)	0.157	0.82	81(0.137)	73(0.104)	0.085	1.36	NC	0.94(0.78–1.13)
AGT/GCG	178(0.377)	158(0.336)	0.197	1.20	255(0.387)	426(0.324)	0.006	1.32	218(0.369)	257(0.367)	0.954	1.01	0.035	1.18(1.04–1.34)
GCG/GCG	51(0.108)	34(0.072)	0.068	1.55	76(0.115)	100(0.076)	0.004	1.59	77(0.130)	78(0.111)	0.304	1.19	0.005	1.42(1.16–1.75)
GCG/Other	46(0.098)	27(0.057)	0.028	1.77	50(0.076)	87(0.066)	0.452	1.16	54(0.091)	52(0.074)	0.309	1.25	0.086	1.32(1.04–1.66)
Other/Other	6(0.013)	7(0.015)	0.789	0.85	9(0.014)	18(0.014)	1.000	1.00	8(0.014)	8(0.011)	0.804	1.19	NC	1.01(0.56–1.72)

a Calculated using a Williams-corrected G test.

b Calculated using Fisher's combined test.

c Allele 1 rs2239657–allele 1 rs7021880–allele 1 rs7021049 / allele 2 rs2239657–allele 2 rs7021880–allele 2 rs7021049.

d P-values calculated using Fisher's exact test.

e Calculated for diplotypes with the same effect (risk or protection) in all three sample sets, with use of Fisher's combined test.

f Mantel-Haenszel common odds ratio with confidence intervals from Monte Carlo simulation.

Assuming a disease prevalence of 1%, we calculated the relative risk of RA in those individuals carrying 2 copies of the protective AGT haplotype compared to those without the AGT haplotype (RR_2 copies AGT_ = 0.77). This homozygous relative risk was substanftially reduced from the relative risk calculated for individuals carrying only one copy of the AGT haplotype (RR_1 copy AGT_ = 1.06.). Similarly, we estimated the relative risks for the susceptible GCG haplotype (RR_2 copies GCG_ = 1.38; RR_1 copy GCG_ = 1.15).

### Genetic Background-Conditioned Results

We used a collection of 749 SNPs informative for European substructure to stratify both the cases and controls in Sample Set 2 [41]. By partitioning cases and controls into similar genetic background groups (“Northern European” or “Other”), our aim was to interrogate the data for strata-specific effects – that is, whether or not association signals were specific to one of these genetic background groups – and avoid potential confounding by population stratification. Although two SNPs demonstrated moderately higher significance levels following stratification – rs16910233 in *C5* (P_North_ = 0.019 compared to P_Unstrat_ = 0.147) and rs12685539 in *CEP110* (P_Other_ = 0.038 compared to P_Unstrat_ = 0.115), a Breslow-Day test of effect heterogeneity comparing OR_North_ and OR_Other_ was not significant. Furthermore, a positional plot of Mantel-Haenszel P-values, testing for association given the genetic background stratification, was very similar to the unadjusted plot (Figure S1) suggesting that stratification of the case and control samples by SNPs informative for European substructure did not change the association patterns in Sample Set 2.

### Rheumatoid Factor (RF)

Rheumatoid factor, a circulating antibody to immunoglobulin G, is a key serum analyte used in diagnosis of RA as well as an aid for the prognosis of RA-severity [2]. As the R620W missense polymorphism in *PTPN22* appears to have stronger susceptibility effects for RF-positive disease [9]–[11] and since RF is clinically important, we investigated the role of RF status on the chr 9q33.2 association patterns for the three LD Block 1 SNPs, rs2239657, rs7021880 and rs7021049, testing for both strata-specific effects as well effect size differences between RF-positive and RF-negative disease.

To explore the effect isolated to RF-positive patients compared to controls, we performed a strata-specific analysis for all sample sets using a genotypic test. The resulting combined P-values for the RF-positive stratum were highly significant (P_rs2239657_ = 4.02E-05, P_rs7021880_ = 7.10E-06, P_rs7021049_ = 5.68E-06; Table 5), which were slightly less significant when compared to the overall genotypic combined P-values (Table 2). A similar analysis of RF-negative disease in Sample Sets 2 and 3 yielded genotypic combined P-values of P_rs2239657_ = 0.038, P_rs7021880_ = 0.013 and P_rs7021049_ = 0.082. Allelic odds ratios and 95% confidence intervals were also calculated for each individual sample set and the results did not demonstrate a clear pattern of strata-specific effects within a stratum or differential effects between the two strata (Table 5). A Breslow-Day test was performed on Sample Set 2 (individually matched cases and controls) to formalize the test of homogeneity of odds ratios, showing that none of the three SNPs exhibited significant differential effects (Table 5). Similarly, results for the analogous Monte Carlo-based test performed in Sample Set 3 (where cases and controls were not individually matched) also did not reveal significant heterogeneity between RF-positive and RF-negative effects.

**Table 5:** Analysis of rs2239657, rs7021880 and rs7021049 stratified by the presence of rheumatoid factor.

	rs2239657						rs7021880						rs7021049					
	Genotypes						Genotypes						Genotypes					
	GG	GA	AA	MAF	P^a^	OR_Allelic_	CC	CG	GG	MAF	P^a^	OR_Allelic_	GG	GT	TT	MAF	P^a^	OR_Allelic_
**Sample Set 1** b																		
RF-positive cases	62	224	184	0.370	0.008	1.35 (1.11–1.63)	51	225	195	0.347	0.001	1.43 (1.17–1.74)	103	229	140	0.461	0.002	1.39 (1.16–1.67)
Controls	45	195	229	0.304			34	187	249	0.271			68	222	180	0.381		
**Sample Set 2**																		
RF-positive cases	68	268	206	0.373	5.60E-04	1.32 (1.14–1.55)	62	250	229	0.346	2.39E-04	1.27(1.08–1.49)	107	283	152	0.458	8.77E-04	1.30 (1.12–1.50)
Matched controls	106	457	520	0.309			87	425	571	0.277			175	505	403	0.395		
RF-negative cases	19	57	41	0.406	0.013	1.63 (1.18–2.27)	15	56	46	0.368	0.005	1.74(1.24–2.44)	26	59	32	0.474	0.054	1.41 (1.02–1.93)
Matched controls	19	100	115	0.295			13	91	130	0.250			29	125	80	0.391		
Breslow-Day^c^					0.263						0.222						0.656	
**Sample Set 3**																		
RF-positive cases	47	156	111	0.398	0.07	1.25 (1.03–1.52)	42	151	121	0.374	0.184	1.19(0.98–1.45)	73	164	77	0.494	0.019	1.28 (1.06–1.55)
RF-negative cases	13	63	46	0.364	0.483	1.09 (0.82–1.44)	12	63	47	0.357	0.312	1.11(0.83–1.47)	21	67	34	0.447	0.297	1.06 (0.81–1.39)
Controls	82	320	298	0.346			79	309	312	0.334			137	331	232	0.432		
**Monte Carlo** ^d^					0.218						0.645						0.116	
**Combined**																		
RF-positive cases^e^					4.02E-05						7.10E-06						5.68E-06	
RF-negative cases f					0.038						0.013						0.082	

a Genotypic P-values were calculated except where indicated.

b All cases in this sample set were RF-positive.

c Differential effects between RF-positive and RF-negative association were determined for sample set 2 using a Breslow-Day test (cases and controls were individually matched).

d Differential effects between RF-positive and RF-negative association were determined for sample set 3 using a Monte Carlo simulation (cases and controls were not individually matched).

e Includes all three sample sets.

f Includes sample sets 2 and 3.

### Logistic Regression

To further dissect association signals from LD patterns, build predictive models and explore the relative effects of each SNP within the models constructed we used logistic regression. To accomplish this we first minimized the number of SNPs for these analyses by calculating pairwise r^2^ values for the 43 SNPs genotyped in all three sample sets and divided the SNPs into distinct groups based on their LD structure. SNPs with pairwise r^2^ values >0.90 were grouped together resulting in 27 distinct groups (Table 6) and then the single most significant SNP from each group (P_comb_ from Table 2) was chosen for the logistic regression analyses.

**Table 6:** Pairwise logistic regression analysis of 27 chr 9q33.2 SNPs.

Group^a^	Marker	r^2^ with rs7021049^b^	P^c^	P adjusted for rs7021049	P adjusted for rs7021049 & rs10985196
3	rs10760112	0.157	0.357	0.285	0.770
4	rs10760117	0.329	0.011	0.760	0.579
5	rs10739575	0.086	0.055	0.580	0.893
6	rs933003	0.011	0.757	0.420	0.448
7	rs1837, rs7026635	0.151	0.002	0.169	0.126
8	rs1056567	0.243	5.22E-04	0.200	0.208
1	rs2239657, rs1953126, rs1609810, rs881375, rs6478486, rs2900180	0.685	2.52E-06	0.217	0.254
9	rs7021880	0.607	1.39E-06	0.104	0.072
2	rs7021049, rs4836834	1	1.24E-06	–	–
10	rs2269066	0.114	0.002	0.115	0.094
11	rs2269067	0.261	7.64E-06	0.023	0.175
12	rs2159776	0.143	0.291	0.367	0.598
13	rs17611, rs7040033, rs2416811, rs9657673, rs3736855	0.328	0.011	0.716	0.450
14	rs10985126	0.206	1.86E-04	0.103	0.992
15	rs1323472, rs7030849	0.585	1.99E-04	0.935	0.415
16	rs12683062	0.113	0.042	0.696	0.327
17	rs3747843	0.337	0.112	0.123	0.059
18	rs10760152, rs10081760	0.297	0.007	0.933	0.790
19	rs942152	0.434	2.92E-05	0.161	0.919
20	rs9408928, rs9409230	0.063	0.270	0.955	0.307
21	rs10985196, rs7046030, rs12683459	0.089	6.17E-06	0.001	–
22	rs306781	0.015	0.905	0.661	0.147
23	rs4837839	0.079	0.171	0.988	0.667
24	rs306783	0	0.192	0.210	0.987
25	rs306784	0.009	0.054	0.144	0.876
26	rs10818527	0.02	0.007	0.044	0.368
27	rs12683989	0.019	0.009	0.054	0.573

a SNPs were grouped together if their pairwise r^2^ values were >0.90. The first SNP in each group was used for the analyses. With the exception of Groups 1 and 2, they are listed in order of appearance on the chromosome (for groups of SNPs we used the position of the first SNP). To avoid confusion, we retained the identity of the Group 1 and 2 SNPs assigned in Figure 2.

b Pairwise LD between rs7021049 and each of the 27 other SNPs as measured by r^2^ in the cases and controls of the combined analysis of all three sample sets.

c Univariate analysis using logistic regression.

In the univariate analysis, the *TRAF1* intronic SNP rs7021049, which marks the Group 2 SNPs in LD Block 1, was the most significant SNP (P = 1.24E-06), followed by rs7021880 (1.39E-06) and then the Group 1 SNP, rs2239657 (P = 2.52E-06) (Table 6). In addition, 11 other SNPs were significant (P<0.01). To assess whether other observed associations in the region were primarily a result of LD with the most significant SNP, we performed pairwise logistic regression on all 27 SNPs adjusting for rs7021049. One SNP retained modest statistical significance (P<0.01): rs10985196 (Group 21), a *GSN* intronic SNP (P = 0.001). To test whether the combination of the rs7021049 and rs10985196 variants fully accounted for the association with RA, we repeated the logistic regression adjusting for both; none of the remaining groups of SNPs were significantly associated with RA. It should be noted, however, that analyses of each individual sample set suggested the evidence for association with rs10985196 (Group 21) was primarily driven by the data from sample set 2 (data not presented).

To explore more complex models, we used both forwards and backwards stepwise logistic regression procedures separately on the same 27 SNPs in each individual sample set as well as in a combined analysis of all three sample sets. The final models generated from the stepwise procedures, however, were inconsistent across the sample sets (Table S2). In fact, seven distinct models were produced; the only instance where the same model was produced was for both the forwards and backwards models of Sample Set 2. Not surprisingly, the forward model for the combined samples, which included two SNPs, rs7021049 (the Group 2 *TRAF1* intronic SNP) and rs10985196 (the *GSN* intronic SNP), was consistent with the results of the pairwise logistic regression analysis presented above.

### Multi-Locus RA Risk Calculations

**Figure 4:**
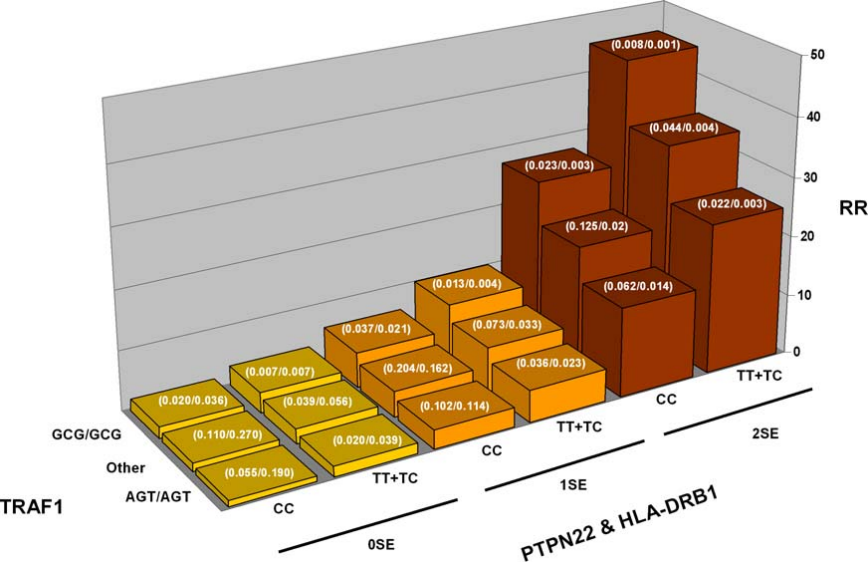
Relative risk plotted as a function of the genetic load of three validated RA risk variants in *HLA-DRB1*, *PTPN22* and *TRAF1*. Individuals are classified according to the number of copies of the *HLA-DRB1* shared epitope (0, 1 and 2) (SE-positive *HLA-DRB1* alleles found in this sample set were: 0101, 0102, 0401, 0404, 0405, 0408 and 1001), carriage of the W620 *PTPN22* missense SNP (TT + CT vs CC) and diplotypes at the TRAF1 SNPs, rs2239657, rs7021880 and rs7021049. The frequency of each combination of markers in cases and controls is highlighted in white (case/control).

Given that we have begun to witness the application of associated genetic variants to disease prognosis [42],[43] and thus far we have convincing evidence for three RA-predisposing loci in our studies (*HLA-DRB1*, *PTPN22* and the *TRAF1* region), we estimated the risk of RA given genotypes at these three loci under three different possible unconditional RA risk assumptions (i.e. RA disease prevalence values) using Bayes' theorem. In total, there were 18 multi-locus genotype combinations and RA risk was calculated for each combination using data from Sample Set 1 as described in the Materials and Methods. Assuming a 1% RA prevalence, similar to that observed in the white North American general population, the results indicate that individuals with the protective genotype at all three loci (0SE for *HLA-DRB1*, CC genotype for *PTPN22* and the AGT/AGT *TRAF1* diplotype) have a substantially reduced predicted risk of RA (0.29% vs. 1%), whereas those individuals in the highest-risk category (*HLA*-2SE, TT or TC genotype at *PTPN22*, and the GCG/GCG *TRAF1* diplotype), have an estimated RA risk of 13.06% – representing more than a 45-fold increase in risk (Table S3). These data are presented as a 3-D plot in Figure 4 where the lowest risk value has been reset to 1 and the other values normalized accordingly. Approximately 19% of the general population will find themselves in the low-risk multi-locus genotype category and only 0.06% in the high risk group. In contrast, when the disease prevalence is increased to 30%, as might be observed in high-risk groups such as an early arthritis clinic, the range of risk drops to 7.88-fold, with the posterior probability of RA calculated to be 11% for the lowest-risk genotype combination and increasing to 86.4% in the highest risk category (Table S3).

## Discussion

We undertook a large-scale, multi-tiered association study of RA using a panel of putative functional SNPs that have been successfully applied to case-control studies of other disease phenotypes [32], [44]–[47]. The initial step of this large-scale RA association study, individual genotyping of 87 prioritized SNPs to evaluate DNA quality prior to constructing DNA pools for our scan, led to the identification of the *PTPN22* R620W SNP [9]. This SNP has been both widely replicated and associated with multiple autoimmune diseases [11].

The present study focuses on variants in the chr 9q33.2 region that were also convincingly correlated with RA status. In particular, three groups of SNPs, represented by rs2239657 (Group 1), rs7021049 (Group 2) and rs7021880 were highly significant and showed a localized effect to a 70 kb region extending from rs10985070, in intron 3 of *PHF19*, across *TRAF1* to rs2900180 in the *TRAF1-C5* intergenic region, but excluding the *C5* coding region (LD Block 1 in Figure 1). Examination of the CEU HapMap data identified 16 additional SNPs that were highly correlated (r^2^>0.95) with either the Group 1 or Group 2 SNPs genotyped in this study (Figure 2C) and all 16 fall within this 70 kb region (no such SNPs were found for rs7021880). Across sample sets, the evidence for association at these sites was stronger, maintaining statistical significance after correction for multiple testing, and more consistent than sites in neighboring regions. Additional analyses further buttressed the statistical support for these conclusions: (i) a haplotype sliding window analysis of all SNPs genotyped in the chr 9q33.2 region demonstrated strong statistical evidence for the *TRAF1*-region harboring RA risk variants (P_comb_ = 4.15E-08) and (ii) haplotype analysis of SNPs within the 70 kb LD Block 1, identified a common protective haplotype (P_comb_ = 3.08E-08) and a less frequent risk haplotype (P_comb_ = 8.00E-09). The three representative SNPs (rs2239657, rs7021049 and rs7021880) were strongly associated with RF-positive disease and trended towards association in RF-negative disease although the small number of RF-negative patients in our study precludes drawing statistically meaningful conclusions about the role of these SNPs in this patient population.

To tease apart association signals from LD patterns, we used logistic regression. The pairwise analyses of the combined datasets suggest there may be two independent statistical signals of association to RA at chr 9q33.2 – one in the *TRAF1* region represented by rs7021049 and one in the *GSN* region represented by rs10985196 (Table 6); however, analyses of the individual sample sets showed rs10985196 was independently associated with disease risk in Sample Set 2 only while rs7021049 showed consistent association across all three sample sets (data not presented). Consequently, additional samples are needed to determine whether the *GSN* region truly contains RA-predisposing effects.

To explore more complex models and assess whether SNPs outside of LD Block 1 were incorporated into these models, we used both forwards and backwards stepwise logistic regression. The sets of SNPs included in the models chosen by the stepwise procedures were inconsistent indicating that the observed association in the region is not clearly explained by a single SNP or set of SNPs included in the tested models.

Independently, Plenge and colleagues [34], using a whole genome association (WGA) study, and Kurreeman and coworkers [35], using a candidate gene approach, have also shown this chr 9q33.2 region is associated with RA risk in whites of European descent. Although a partially overlapping subset of samples was used in all three studies (see Table 1 footnotes), each study employed unique experimental designs, analyses and presented different facets of the 9q33.2-RA association. Plenge and colleagues [34] identified rs3761847, a *TRAF1/C5* intergenic SNP, as one of two non-MHC SNPs reaching genome-wide significance in their WGA study; not surprisingly, the other significant non-MHC SNP was in *PTPN22*. Their follow-up fine mapping of the chr 9q33.2 region with nine haplotype tag SNPs in four RA sample sets (2519 cases / 3627 controls) localized the region of interest to 100 kb extending from *PHF19* into *C5*. rs3761847, which is a Group 2 SNP in LD Block 1, remained the most significant SNP in their combined analysis (P = 4.00E-14) followed by rs2900180 (P = 8.00E-14), a member of the Group 1 SNPs in LD Block 1.

Taking a candidate gene approach, Kurreeman and colleagues [35] studied 40 SNPs in a 300 kb region surrounding *C5* (from the 3′ UTR of *PHF19* to intron 25 of *CEP110*) in a staged-approach in four sample sets (2,719 cases / 1999 controls) and concluded that rs10818488, another *TRAF1-C5* intergenic SNP and member of the LD Block 1 Group 2 SNPs, was the SNP most significantly associated with a diagnosis of RA in their study. Association of a Group 1 SNP, rs2416806, was moderately significant in a combined analysis of three of their sample sets (P = 0.015). Neither the Plenge et al. nor Kurreeman et al. study included rs7021880.

Our analyses, which included more SNPs and incorporated HapMap information for all SNPs highly correlated with SNPs genotyped in our study, permitted a comprehensive analysis of the genetic architecture of 9q33.2 region, allowing us to localize the RA-susceptibility effects to a 70 kb region (LD Block 1) that includes a portion of *PHF19*, all of *TRAF1* and the majority of the *TRAF1-C5* intergenic region, but excludes the *C5* coding region, narrowing the true region of interest. Our data, however, did not allow us to identify a single SNP or group of highly correlated SNPs (r^2^>0.95) in this 70 kb region that unambiguously explained the association signal in all three independent sample sets. Other sample sets with different patterns of LD or functional studies will be required to resolve this issue.

Interestingly, Potter and colleagues [48], who studied 23 haplotype-tagging SNPs from the 6 *TRAF* genes, including three from *TRAF1*, in a UK case-control study (351 RA cases / 368 controls) failed to see association with both a Group1 (rs1468671) and a Group2 (rs4836834) SNP. Using an overlapping sample set to the Potter et al study, the recent Welcome Trust Case Control Consortium genome-wide association study of RA (1860 cases / 2930 controls) [25] also failed to identify RA-risk variants in this region. However, a more recent follow-up study from the same group of an independent RA sample set from the UK (3418 cases / 3337 controls) confirmed association with four LD Block 1 Group 2 SNPs although the effect size was less (meta analysis OR 1.08, 95%CI 1.03–1.14) [49].

The original RA-associated, 9q33.2 SNP identified in our genome-wide scan, rs1953126, is located within LD Block 1, 1 kb upstream of the 5′ end of *PHF19*, the human homologue of the Drosophila polycomblike protein, *PCL*, gene. In Drosophila, the protein encoded by this gene is part of the 1MDa extra sex combs and enhancer of zeste [ESC-E(Z)] complex which is thought to mediate transcriptional repression by modulating the chromatin environment of many developmental regulatory genes such as homeobox genes. While the exact function of this gene in humans remains unknown, it encodes two nuclear proteins that appear to be upregulated in multiple cancers and preliminary evidence suggests that deregulation of these genes may play a role in tumor progression [50].

*TRAF1* encodes a protein that is a member of the TNF receptor (TNFR) associated factor (TRAF) protein family that associates with, and mediates signal transduction from various receptors including a subset of the TNFR superfamily. There are six members of this family of adaptor proteins; however, TRAF1 is unique in that while it contains the hallmark carboxyl-terminal TRAF domain, it has a single zinc finger in the amino-terminal part and the N-terminal RING finger domain, required for NF-κB activation, is missing. TRAF1 appears to have both anti-apoptotic and anti-proliferative effects [51],[52]. In addition, this protein has been found to be elevated in malignancies of the B cell lineage [53]–[58]. This observation is interesting given the risk of lymphoma, particularly diffuse large B cell lymphomas, appears to be increased in the subset of RA patients with very severe disease, independent of treatment [59],[60]. Although the precise mechanism of TRAF1 action in various signaling pathways has not been fully elucidated, it is clear that this molecule plays an important role in immune cell homeostasis making it an excellent candidate gene for RA. In fact, *in vitro* work suggests that TNFα-mediated synovial hyperplasia, a major pathophysiologic feature of RA, may be correlated with upregulation of TRAF molecules, particularly TRAF1 [61]. Given that TNF blockade has proved a highly effective therapy for RA [62],[63] and response to TNF-antagonists among RA patients is known to vary, investigation of whether the *TRAF1* variants identified in this study play a role in this differential response may be a fruitful pharmacogenetic avenue to pursue.

*C5* is also an excellent RA candidate gene and although our analyses allowed us to exclude the *C5* coding region, SNPs in LD Block 1 could differentially regulate the expression of this gene. *C5* encodes a zymogen that is involved in all three pathways of complement activation. Traditionally, the complement system has been viewed as a central part of the innate immune system in host defenses against invading pathogens and in clearance of potentially damaging cell debris; however, complement activation has also recently been implicated in the pathogenesis of many inflammatory and immunological diseases. Proteolytic cleavage of C5 results in C5a, one the most potent inflammatory peptides, and C5b, a component of the membrane attack complex (MAC) that can cause lysis of cells and bacteria. Genetic studies in various mouse models of RA, including collagen-induced arthritis (CIA) and the K/BXN T cell receptor transgenic mouse model of inflammatory arthritis have provided evidence that *C5* or a variant in strong LD, plays a role in disease [64]–[66]. More striking is the observation that anti-C5 monoclonal antibody therapy can prevent and ameliorate disease in both mouse models [67],[68].

In summary, we have independently identified a region on chr 9q33.2 as a risk locus for RA. Although the evidence from the SNPs genotyped in our sample sets most strongly points towards *TRAF1* variants as being the most highly consistent with a disease model, the high LD that extends from the 5′ end of *PHF19* through *TRAF1* and into the *TRAF1-C5* intergenic region precludes conclusively determining causative genes or functional motifs through genetic means in these samples. Mapping studies in additional sample sets with a different LD architecture and/or functional studies will be required to resolve the molecular relevance of these findings.

Aside from the possibility of developing targeted therapies with knowledge of predisposing variants underlying the onset of RA, the identification of RA susceptibility alleles may encourage earlier monitoring and provide an intervention avenue in advance of significant joint erosion. Our initial analysis of the three known genetic risk factors, *HLA-SE*, *PTPN22* and the chr 9q33.2 variants described here, suggests a >45 fold difference in RA risk depending on an individual's genotype at these three loci. As additional markers are identified, the ability to accurately predict individuals at increased risk for developing RA, particularly within families with a history of RA, may prove useful. Finally, differential risk variants may prove to be drug response markers.

## Materials and Methods

### Subjects and Samples

All RA cases included in this study were white and met the 1987 American College of Rheumatology diagnostic criteria for RA [69]; informed written consent was obtained from every subject. Sample Set 1, which consisted of 475 RA cases and 475 individually-matched controls, was collected by Genomics Collaborative, Inc. All case samples were white North Americans of European descent who where rheumatoid factor (RF) positive. Control samples were healthy white individuals with no medical history of RA, also of European descent. A single control was matched to each case on the basis of sex, age (±5 years), and self-reported ethnic background. The 661 cases in Sample Set 2 were acquired from the North American Rheumatoid Arthritis Consortium (NARAC) (http://www.naracdata.org/) and consisted of members from 661 white North American multiplex families [33],[70],[71]. Both RF-positive and RF-negative patients were included in this sample set. Controls for Sample Set 2 were selected from 20,000 healthy individuals enrolled in the New York Cancer Project [72], a population-based prospective study of the genetic and environmental factors that cause disease (http://www.amdec.org/). Two control individuals were matched to a single, randomly chosen affected sibling from each NARAC family on the basis of sex, age (decade of birth), and self-reported ethnic background. Sample Set 3 was composed of 596 white RA patients from the Leiden University Medical Centre and 705 white controls from the same geographic region in The Netherlands [73]–[75]. Both RF-positive and RF-negative patients were included in this sample set. Table 1 displays the clinical characteristics of all three sample sets and a detailed description of samples that overlap with published studies of this region [34],[35].

### Functional Genome-Wide Scan

Our functional genome-wide scan included 25,966 gene-centric SNPs curated from dbSNP, the Applera Genome Initiative [44],[76] and the literature. SNPs were included if they appeared in more than one database and had a minor-allele frequency >1%. Approximately seventy percent of the SNPs were annotated as missense polymorphisms. The majority of the remaining SNPs were either located within putative transcription-factor site motifs or within acceptor/donor splice site regions or were nonsense polymorphisms.

### Genotyping

Allele-specific, real-time quantitative PCR [77] was used to amplify 3 ng of pooled DNAs and infer SNP allele frequencies as previously detailed [44]. Individual genotyping on SNPs was performed on 0.3 ng of DNA using a similar protocol. Blinded to case-control status, custom-made in-house software was used to call genotypes, followed by hand-curation. Individual genotyping accuracy has been estimated to be >99.8% by comparison with an independent method. *HLA-DRB1* genotyping was performed using sequence-specific oligonucleotide probes as previously described [9]. Shared epitope (SE) status [78] was determined from the probe hybridization patterns. For this study, *DRB1* alleles positive for the SE include: 0101, 0102, 0401, 0404, 0405, 0408 and 1001.

### Fine-Scale Mapping SNP Selection

To identify SNPs for inclusion in our fine-scale mapping effort of the 9q33.2 region, we first postulated two different disease models: 1) a model where the originally identified SNP is in linkage disequilibrium with one or more causative SNPs and 2) a model of allelic heterogeneity where several alleles at the locus independently predispose individuals to RA. To address both of these models, we first defined the region to be interrogated by calculating pairwise linkage disequilibrium (r^2^) values between the originally identified SNP 5′ of *PHF19*, rs1953126, and all HapMap-genotyped SNPs (http://www.hapmap.org/) within 500 kb flanking either side for the CEPH samples (Utah residents with ancestry from northern and western Europe, or CEU individuals) [36]. With this information, we defined a broad region spanning 668 kb from *MEGF9*, 177 kb upstream of rs1953126 , to *STOM*, 491 kb downstream of rs1953126, for follow-up genotyping. SNPs within this region were partitioned into those in moderate to high LD (r^2^>0.20) with rs1953126 to address the first model, and those in low LD (r^2^<0.20) with rs1953126 to address the second model. The power-based SNP selection program Redigo [79] was then used on the low LD set of SNPs to identify a reduced number of SNPs (tagging SNP set) that retained high power to detect association. Those SNPs in moderate to high LD with the original SNP were reduced by selecting a subset of representative SNPs of any groups exhibiting extremely high inter-group LD (r^2^>0.98). Further, any putative functional SNPs were automatically included in the fine-scale mapping effort if we were able to construct high-quality genotyping assays for them. The resulting set of 137 SNPs was genotyped in Sample Set 1 and the data analyzed. Additional removal of fine-scale mapping SNPs was performed for evaluation in subsequent sample sets on the basis of association results and refined LD patterns: a subset of 72 SNPs were selected for genotyping in Sample Set 2 and 42 SNPs were genotyped in Sample Set 3.

### Statistical Analyses

The Cochran-Armitage trend test [80] was used to calculate P-values for individual SNPs. A William's-corrected G-test [37] was used to calculate P-values for genotypic association. P-values were corrected for multiple testing using the method of Dunn-Sidak [37]. Odds ratios and confidence intervals were calculated according to standard procedures. Hardy-Weinberg equilibrium testing was accomplished through the exact test of Weir [81]. P-values were combined across sample sets using the Fisher's combined P-value, or omnibus procedure [82]. Likewise, Mantel-Haenszel common odds ratios [83] were calculated to combine data across sample sets. To avoid the small-count limitations of asymptotic-derived confidence intervals, a Monte Carlo simulation was written in XLISP-STAT to calculate 95% confidence intervals on the Mantel-Haenszel common odds ratios. We typically performed 20,000 iterations of the Monte Carlo for these confidence intervals. The standard measure of pairwise linkage disequilibrium (the r^2^ statistic from estimated 2-site haplotypes) was used to characterize the genetic architecture of the region. The program LDMAX with an EM algorithm was used to perform the r^2^ calculations [84].

### Genetic Analyses

#### Haplotype Analysis

Haplotypes were estimated from unphased genotype data and evaluated for association with RA through the Haplo.Stats software package [39]. A sliding window of haplotype association was calculated using a window size of 5 SNPs. Global P-values (calculated across all haplotypes within a window) and haplotype-specific ORs and P-values were calculated. Additional haplotype analyses were performed using a combination of the Pseudo-Gibbs sampling algorithm in the program SNPAnalyzer (http://snp.istech.info/snp/SNPAnalyzer.html) [40] and the Haplo.Stats package.

#### Genetic Background-Conditioned Analysis

A panel of 749 SNPs previously selected to be informative for classifying individuals of European descent into northern and southern geographical groups was applied to case and control samples from the second sample set as described previously [41]. Applying this method, we placed 367 cases and 525 controls from Sample Set 2 into a northern European ancestry cluster. Each case or control individual had a greater than 0.95 probability of belonging to the northern European cluster. The remaining cases and controls from this study were binned into an “Other” category. A Breslow-Day analysis [85] was applied to the stratified data to test for heterogeneity in ORs between the two groups for the 9q33.2-linked SNPs studied here. To test for association conditioned on these stratified data, we also calculated a Mantel-Haenszel P-value [83].

#### Subphenotype Analysis: Rheumatoid Factor

Rheumatoid Factor (RF) levels were measured in cases as previously described [86],[87]. To test for heterogeneity of effect between RF-positive and RF-negative patients we used two different methods. In sample set 2, where case-control matching was part of the study design, we used the Breslow-Day [85] test. Since individual matching was not incorporated into Sample Set 3, we used a Monte Carlo simulation to compare the effect size for RF-positive patients versus all controls to the effect size for RF-negative patients versus all controls. Similar to other tests of homogeneity of odds ratios, we constructed a test statistic measuring the departure between normalized odds ratios comparing two groups (see equation S1 in Text S1) and ran a Monte Carlo simulation to account for correlated odds ratios in the null distribution. Monte Carlo P-values were calculated in the traditional manner.

#### Logistic Regression

Logistic regression models were performed to assess the relative importance of 27 SNPs chosen as distinct representatives of groups of SNPs with pairwise r^2^ values >.90. First, a logistic regression model for each unique pair of SNPs was performed. These pairwise models assumed a multiplicative effect on the risk of RA for each additional copy of an allele. P-values and odds ratios for the effect of each SNP, when controlling for each alternative SNP, were examined visually to determine if any SNP showed obvious patterns (attenuating the risk of each alternate SNP and retaining risk when adjusted for each alternate SNP). These types of patterns might be expected under a disease model of a single functional SNP. For models in which both SNPs remained strongly associated (P<.01), additional models were performed to determine if adding a third SNP significantly improved the model. To examine multi-SNP relationships in a more automated fashion in a manner similar to that suggested by Cordell and Clayton [88], both a forward as well as a backward stepwise logistic regression procedure was performed on each sample set individually as well as on the combined sample sets. The stepwise models were performed coding the genotypes with indicator variables and with a significance level of 0.05 for the two degree of freedom score test (for entry) or Wald test (for exit) on the effect of the SNP used as a threshold for entry or exit from the model. Models applied to the combined sample sets also forced sample set as a covariate in the model. The final model from each procedure was also applied to the other sample sets to assess consistency of the models across sample sets. The P-value from the likelihood ratio test of the global null hypothesis for each model is reported for single studies while for the combined study we report a P-value from a likelihood ratio test comparing the model containing the SNPs and the variable representing sample set to a model containing only the same sample set variable. All logistic regression models were performed using SAS version 9.

#### Multi-Locus RA Risk Calculations

Risk for RA given every possible 3-locus genotype combination at the *HLA-DRB1* shared epitope, the R620W SNP in *PTPN22*, and 3-SNP *TRAF1* diplotypes was calculated for sample set 1 using Bayes' theorem (see equations S2 and S3 in Text S1) assuming conditional independence between loci (the commonly-used Naive Bayes model for predictive modeling) and a range of RA prevalence values (1%, 10% and 30%). Theoretical calculations (not shown) demonstrate that unless both sample sizes and epistatic effects are very large, probability estimates of the jointly-occurring genotypes have lower error rates assuming conditional independence between loci. Therefore, fully-factorizing the probability of multi-locus genotypes (using the conditional independence assumption) is warranted under a broad range of the parameter space. By estimating the posterior probability of RA for every possible multi-locus genotype combination, accurate individual-based prognosis is possible. Confidence intervals on the relative risk estimates were obtained through simulation. It is important to note that due to the selection of loci for inclusion in the model, some overfitting may be present.

## Supporting Information

Figure S1 Genetic background-conditioned analysis. Allelic association for individual SNPs genotyped in Sample Set 2 (in blue) plotted as a function of position along with a Mantel-Haenszel P-value using stratifying information from an ancestry clustering procedure (in red).
(0.41 MB TIF)

Table S1 Minor allele frequencies and allele-based association of chr 9q33 SNPs with RA.
(0.10 MB XLS)

Table S2 Log likelihood ratio P-values for forward and backwards models using logistic regression.
(0.05 MB DOC)

Table S3 RA risk estimates for 3 loci – *HLA-SE*, *PTPN22*, and *TRAF1* – assuming a disease prevalence of 1%, 10% and 30%.
(0.11 MB DOC)

Text S1 Rheumatoid factor analysis and multilocus RA risk calculations.
(0.06 MB DOC)
