# Supplementary figures and images for: A Large-Scale Rheumatoid Arthritis Genetic Study Identifies Association at Chromosome 9q33.2
Source: PLoS Genet. 2008 Jun 27;4(6):e1000107. doi: 10.1371/journal.pgen.1000107 (PMC2481282; doi:10.1371/journal.pgen.1000107)

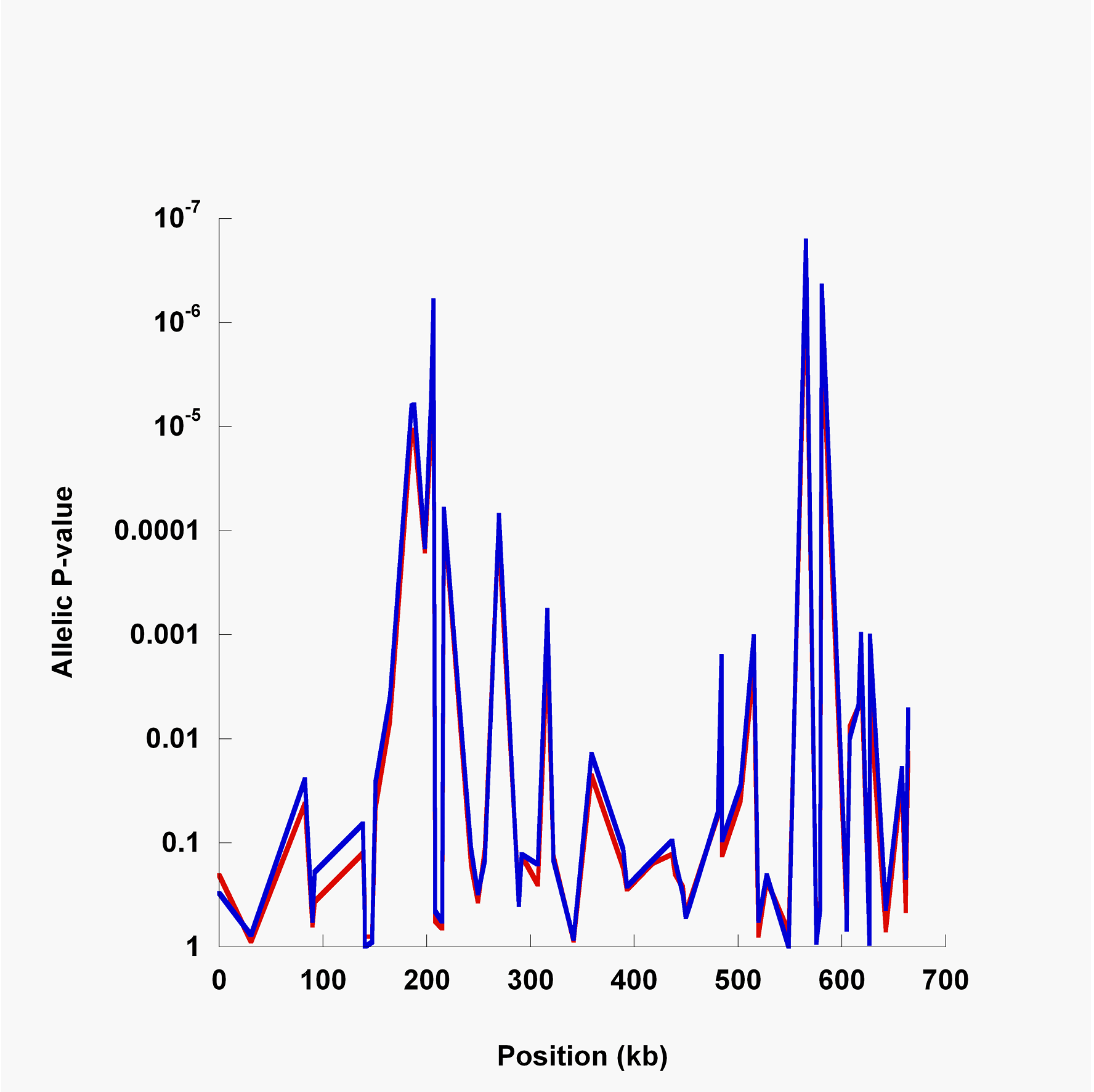

Supplement: Figure S1 — Genetic background-conditioned analysis. Allelic association for individual SNPs genotyped in Sample Set 2 (in blue) plotted as a function of position along with a Mantel-Haenszel P-value using stratifying information from an ancestry clustering procedure (in red). (0.41 MB TIF) [file pgen.1000107.s001.tif]
