# Supplementary material for: A Large-Scale Rheumatoid Arthritis Genetic Study Identifies Association at Chromosome 9q33.2
Source: PLoS Genet. 2008 Jun 27;4(6):e1000107. doi: 10.1371/journal.pgen.1000107 (PMC2481282; doi:10.1371/journal.pgen.1000107)
Supplement: Text S1 — Rheumatoid factor analysis and multilocus RA risk calculations. (0.06 MB DOC) [file pgen.1000107.s005.doc]

**SUPPORTING INFORMATION**

*Rheumatoid Factor Analysis*

Investigating the effect heterogeneity between two case groups, RF-positive and RF-negative disease, with the same group of controls, we devised a Monte Carlo procedure using a simple test statistic to measure the normalized departure between two odds ratios. As the correlated nature of the two odds ratios was automatically incorporated into the Monte Carlo simulation, we were able to obtain the appropriate null distribution of this test statistic without complicated analytic techniques. The test statistic constructed was

$T=\left(\frac{\ln OR_{1}}{\sqrt{\frac{1}{x_{1}}+\frac{1}{x_{2}}+\frac{1}{z_{1}}+\frac{1}{z_{2}}}}-\frac{\ln OR_{2}}{\sqrt{\frac{1}{y_{1}}+\frac{1}{y_{2}}+\frac{1}{z_{1}}+\frac{1}{z_{2}}}}\right)^{2}$ ; (eqn S1)

where $OR_{1}$ is the allelic odds ratio comparing RF-positive cases to the control group; $OR_{2}$ is the allelic odds ratio comparing RF-negative cases to the control group; and $x_{1}$ and $x_{2}$ are the allelic counts for the A_1_ allele and A_2_ allele in the RF-positive case group, respectively. Using similar notation, $y_{1}$ and $y_{2}$ are the allelic counts in RF-negative cases, and $z_{1}$ and $z_{2}$ are the allelic counts in the control group.

*Multilocus RA Risk Calculations*

The probability of RA given the genotypes at the three predisposing loci is

$P\left(RA\text{ }\left|\text{ }G_{HLA},G_{PTPN22},G_{TRAF1}\right.\right)=\frac{P\left(G_{HLA},G_{PTPN22},G_{TRAF1}\text{ }\left|\text{ }RA\right.\right)P\left(RA\right)}{P\left(G_{HLA},G_{PTPN22},G_{TRAF1}\right)}$ (eqn S2)

Assuming conditional independence, we can fully factorize

$\approx\frac{P\left(RA\right)\prod P\left(G\text{ }\left|\text{ }RA\right.\right)}{P\left(RA\right)\prod P\left(G\text{ }\left|\text{ }RA\right.\right)+\left[1-P\left(RA\right)\right]\prod P\left(G\text{ }\left|\text{ }CT\right.\right)}$ (eqn S3)

where $P\left(RA\right)$ is the probability of RA, and where $P\left(G\left|RA\right.\right)$ and $P\left(G\left|CT\right.\right)$ are the probabilities of a genotype in RA patients and controls, respectively.
