# Supplementary material for: A Large-Scale Rheumatoid Arthritis Genetic Study Identifies Association at Chromosome 9q33.2
Source: PLoS Genet. 2008 Jun 27;4(6):e1000107. doi: 10.1371/journal.pgen.1000107 (PMC2481282; doi:10.1371/journal.pgen.1000107)
Supplement: Supplementary file 4 [file pgen.1000107.s003.doc]

Table S2. Log likelihood ratio P-values for forwards and backwards models using logistic regression.

| | | | Tested Sample Sets | | | |
|---|---|---|---|---|---|---|
| Building Sample Set | Model | SNPS[a] | 1 | 2 | 3 | Combined |
| 1 | Forward | rs10760117 | 0.0022 | 0.135 | 0.165 | 3.33E-04 |
| 2 | Forward | rs7021880, rs12683062, rs10985196, rs4837839, rs12683989 | 0.067 | 6.40E-10 | 0.419 | 1.29E-07 |
| 3 | Forward | rs2159776, rs1323472 | 0.051 | 0.084 | 0.0048 | 1.10E-04 |
| Combined | Forward | rs7021049, rs10985196 | 0.010 | 8.25E-08 | 0.089 | 1.57E-09 |
| 1 | Backward | rs10985126, rs2269066, rs10760152, rs306781, rs1323472, rs1837 | 1.25E-04 | 5.82E-04 | 0.077 | 4.70E-06 |
| 2 | Backward | Same Model as Sample Set 2-Forward | 0.067 | 6.40E-10 | 0.419 | 1.29E-07 |
| 3 | Backward | rs2159776, rs3747843, rs2269066, rs2269067, rs1323472 | 0.0963 | 0.0018 | 0.0037 | 6.45E-07 |
| Combined | Backward | rs10818527, rs3747843, rs7021880, rs2269067, rs1323472 | 0.023 | 1.43E-06 | 0.063 | 1.63E-10 |

[a] SNPs included in each model.
