# Supplementary material for: A Large-Scale Rheumatoid Arthritis Genetic Study Identifies Association at Chromosome 9q33.2
Source: PLoS Genet. 2008 Jun 27;4(6):e1000107. doi: 10.1371/journal.pgen.1000107 (PMC2481282; doi:10.1371/journal.pgen.1000107)
Supplement: Supplementary file 5 [file pgen.1000107.s004.doc]

Table S3. RA risk estimates for 3 loci - *HLA-SE*, *PTPN22* and *TRAF1* - assuming a disease prevalence of 1%, 10% and 30%.

| Loci | | | Disease Prevalence | | | | | | | | | | | |
|---|---|---|---|---|---|---|---|---|---|---|---|---|---|---|
| | | | 1% | | | | 10% | | | | 30% | | | |
| *HLA*[a] | *PTPN22*[b] | *TRAF1*[c] | P(MLG)[d] | P(RA\|MLG)[e] | RR[f] | RR[g] | P(MLG)[d] | P(RA\|MLG)[e] | RR[f] | RR[g] | P(MLG)[d] | P(RA\|MLG)[e] | RR[f] | RR[g] |
| 0SE | CC | AGT/AGT | 0.189 | 0.003 | 0.29 (0.21-0.38) | 1.00 | 0.176 | 0.031 | 0.31 (0.23-0.40) | 1.00 | 0.149 | 0.110 | 0.37 (0.27-0.46) | 1.00 |
| 0SE | CC | Other | 0.269 | 0.004 | 0.41 (0.33-0.50) | 1.41 | 0.254 | 0.043 | 0.43 (0.35-0.52) | 1.39 | 0.222 | 0.148 | 0.49 (0.41-0.59) | 1.35 |
| 0SE | TT+TC | AGT/AGT | 0.039 | 0.005 | 0.50 (0.27-0.85) | 1.73 | 0.037 | 0.053 | 0.53 (0.29-0.86) | 1.70 | 0.033 | 0.176 | 0.59 (0.34-0.89) | 1.61 |
| 0SE | CC | GCG/GCG | 0.036 | 0.006 | 0.56 (0.30-0.94) | 1.92 | 0.034 | 0.058 | 0.58 (0.32-0.94) | 1.87 | 0.031 | 0.192 | 0.64 (0.38-0.97) | 1.75 |
| 0SE | TT+TC | Other | 0.055 | 0.007 | 0.71 (0.46-1.05) | 2.44 | 0.054 | 0.073 | 0.73 (0.49-1.05) | 2.35 | 0.051 | 0.232 | 0.77 (0.55-1.04) | 2.11 |
| 0SE | TT+TC | GCG/GCG | 0.007 | 0.010 | 0.96 (0.69-1.16) | 3.33 | 0.007 | 0.097 | 0.97 (0.71-1.14) | 3.12 | 0.007 | 0.292 | 0.97 (0.36-1.96) | 2.66 |
| 1SE | CC | AGT/AGT | 0.114 | 0.009 | 0.90 (0.29-3.26) | 3.09 | 0.113 | 0.090 | 0.90 (0.31-2.70) | 2.92 | 0.110 | 0.277 | 0.92 (0.76-1.11) | 2.53 |
| 1SE | CC | Other | 0.162 | 0.013 | 1.26 (1.05-1.53) | 4.35 | 0.166 | 0.123 | 1.23 (1.04-1.46) | 3.98 | 0.175 | 0.351 | 1.17 (1.03-1.32) | 3.20 |
| 1SE | TT+TC | AGT/AGT | 0.024 | 0.015 | 1.55 (0.92-2.70) | 5.34 | 0.025 | 0.147 | 1.47 (0.94-2.34) | 4.76 | 0.027 | 0.400 | 1.33 (0.95-1.80) | 3.65 |
| 1SE | CC | GCG/GCG | 0.022 | 0.017 | 1.71 (1.00-3.09) | 5.92 | 0.023 | 0.161 | 1.61 (1.00-2.60) | 5.20 | 0.026 | 0.425 | 1.42 (1.00-1.91) | 3.88 |
| 1SE | TT+TC | Other | 0.034 | 0.022 | 2.17 (1.48-3.37) | 7.49 | 0.037 | 0.196 | 1.96 (1.41-2.77) | 6.33 | 0.045 | 0.485 | 1.62 (1.30-1.99) | 4.42 |
| 1SE | TT+TC | GCG/GCG | 0.005 | 0.029 | 2.94 (1.11-13.91) | 10.15 | 0.005 | 0.250 | 2.50 (1.10-6.40) | 8.07 | 0.007 | 0.563 | 1.88 (1.07-2.93) | 5.13 |
| 2SE | CC | AGT/AGT | 0.014 | 0.043 | 4.29 (2.62-8.45) | 14.82 | 0.019 | 0.330 | 3.30 (2.26-5.04) | 10.67 | 0.028 | 0.656 | 2.19 (1.76-2.65) | 5.97 |
| 2SE | CC | Other | 0.021 | 0.060 | 5.95 (3.98-10.07) | 20.55 | 0.030 | 0.410 | 4.10 (3.12-5.49) | 13.26 | 0.051 | 0.729 | 2.43 (2.12-2.75) | 6.64 |
| 2SE | TT+TC | AGT/AGT | 0.003 | 0.072 | 7.23 (2.75-100) | 24.95 | 0.005 | 0.462 | 4.62 (2.39-10) | 14.90 | 0.009 | 0.768 | 2.56 (1.83-3.33) | 7.00 |
| 2SE | CC | GCG/GCG | 0.003 | 0.080 | 7.96 (2.94-100) | 27.48 | 0.005 | 0.488 | 4.88 (2.58-10) | 15.74 | 0.009 | 0.786 | 2.62 (1.91-3.33) | 7.16 |
| 2SE | TT+TC | Other | 0.004 | 0.099 | 9.91 (4.62-34.44) | 34.20 | 0.008 | 0.547 | 5.47 (3.47-8.57) | 17.68 | 0.016 | 0.824 | 2.75 (2.25-3.19) | 7.50 |
| 2SE | TT+TC | GCG/GCG | 0.0006 | 0.131 | 13.06 (NC)[h] | 45.10 | 0.001 | 0.623 | 6.23 (NC)[h] | 20.12 | 0.003 | 0.864 | 2.88 (NC)[h] | 7.88 |

[a] The number of copies of the *HLA-DRB1* shared epitope (SE). SE-positive HLA-DRB1 alleles include: 0101, 0102, 0401, 0404, 0405, 0408 and 1001.

[b] The *PTPN22* R620W genotype (CC indicates homozygosity for the protective R620 allele; TT+TC indicates carriage of the risk W620 allele).

[c] The *TRAF1* diplotype (allele 1 rs2239657- allele 1 rs7021880 - allele 1 rs7021049 / allele 2 rs2239657 - allele 2 rs7021880 - allele 2 rs7021049).

[d] Probability of the indicated 3-locus genotype.

[e] Probability of RA given the indicated 3-locus genotype.

[f] Relative risk and 95% confidence intervals from Monte Carlo simulations using 10,000 replicates.

[g] Standardized relative risk estimates setting the lowest value in each group (disease prevalence) to 1.

[h] 95% CIs were not calculated due to small counts.
